# Perforation of the gastrointestinal tract by a ventriculoperitoneal shunt catheter with anal exteriorization in an adult patient: case report

**DOI:** 10.1093/jscr/rjag437

**Published:** 2026-06-10

**Authors:** Victor Manoel da Silva Correia, João Victor Costa Muller, Henrique Canevari Barros, Maria Vitoria Yuka Messias Nakata, Isadora Delis, Paulo Figueiredo Costa

**Affiliations:** UNESP, Department of General and Gastrointestinal Surgery, Hospital das Clínicas, Botucatu Medical School, Av. Professor Mário Rubens Guimarães Montenegro, s/n, Distrito de Rubião Junior, 18618-687, São Paulo State University, Botucatu, São Paulo, Brazil; UNESP, Department of General and Gastrointestinal Surgery, Hospital das Clínicas, Botucatu Medical School, Av. Professor Mário Rubens Guimarães Montenegro, s/n, Distrito de Rubião Junior, 18618-687, São Paulo State University, Botucatu, São Paulo, Brazil; UNESP, Department of General and Gastrointestinal Surgery, Hospital das Clínicas, Botucatu Medical School, Av. Professor Mário Rubens Guimarães Montenegro, s/n, Distrito de Rubião Junior, 18618-687, São Paulo State University, Botucatu, São Paulo, Brazil; UNESP, Department of General and Gastrointestinal Surgery, Hospital das Clínicas, Botucatu Medical School, Av. Professor Mário Rubens Guimarães Montenegro, s/n, Distrito de Rubião Junior, 18618-687, São Paulo State University, Botucatu, São Paulo, Brazil; UNESP, Department of General and Gastrointestinal Surgery, Hospital das Clínicas, Botucatu Medical School, Av. Professor Mário Rubens Guimarães Montenegro, s/n, Distrito de Rubião Junior, 18618-687, São Paulo State University, Botucatu, São Paulo, Brazil; UNESP, Department of General and Gastrointestinal Surgery, Hospital das Clínicas, Botucatu Medical School, Av. Professor Mário Rubens Guimarães Montenegro, s/n, Distrito de Rubião Junior, 18618-687, São Paulo State University, Botucatu, São Paulo, Brazil

**Keywords:** ventriculoperitoneal shunt, intestinal perforation, hydrocephalus, case report

## Abstract

Ventriculoperitoneal shunt (VPS) is widely utilized in the management of hydrocephalus, although it is associated with various complications, predominantly infectious or mechanical. Intestinal perforation with anal exteriorization of the catheter is a rare but potentially lethal event. We report a 29-year-old male with a history of previously treated arteriovenous malformation and VPS implantation since 2018 presented with anal exteriorization of the catheter. He reported prior episodes of catheter protrusion, inadvertent manual traction, and the discharge of foul-smelling fluid. Imaging suggested distal migration of the catheter into the gastrointestinal tract. He underwent exploratory laparotomy with catheter removal and sigmoid repair, in conjunction with neurosurgery for the removal of the proximal portion of the VPS. The patient had an uneventful postoperative recovery. This case underscores the critical importance of prompt assessment and surgical intervention in the rare complication of intestinal perforation caused by a VPS catheter, especially in cognitively impaired patients.

## Introduction

Ventriculoperitoneal shunt (VPS) is a standard treatment for hydrocephalus, diverting cerebrospinal fluid to the peritoneal cavity. Complications associated with VPS can be significant, with estimates suggesting that ~23.8% of patients experience some form of complication within a 4-year follow-up period [1–3]. The most common complications include shunt infection, which affects 6.1% of cases, and the need for surgical revision in ~22.0% of patients. Less frequently, catheters may migrate to cavities other than the peritoneal cavity, leading to serious complications such as intestinal perforation [4, 5].

Intestinal perforation caused by a VPS catheter is considered an uncommon event, with reported incidences ranging from 0.1% to 0.7%. Although rare, this complication poses significant risks, with an overall mortality rate estimated at around 15% [4, 5]. Cases of gastrointestinal perforation with anal exteriorization have an incidence of less than 0.1% but can lead to severe infections such as meningitis and peritonitis. This report describes a unique instance of anal exteriorization of a VPS catheter in a young adult with partial cognitive impairment, reinforcing the necessity for continuous surveillance and multidisciplinary management [6, 7].

## Case report

### Patient data

Age: 29-years-old; male; farm worker; partially dependent on daily living activities due to confusion and forgetfulness. He holds Brazilian nationality and is single, having completed education from the fifth to the eighth grade.

### History of the current illness

Was referred via state regulation from another location after discovering, 2 months earlier, that the distal end of the VPS catheter had become externalized in the anal region. The device had been previously inserted by the neurosurgery team at the UNESP Clinical Hospital. The patient reported that, in July 2025, he noticed the catheter emerging from the anus for the first time and manually reinserted the device. During the same period, there were reports of three tonic seizures, similar to those that occurred before the catheter extrusion. In October 2025, the catheter protruded from the anus again when the patient pulled on the device and noticed a foul-smelling liquid coming out, which prompted him to seek medical attention. He was then referred to the UNESP Clinical Hospital in Botucatu-SP for management and treatment.

### Relevant personal background

The patient has a history of arteriovenous malformation of the corpus callosum/quadrigeminal plate, with two prior episodes of bleeding accompanied by sudden headaches. He has undergone multiple embolizations for the arteriovenous malformation and had the VPS implanted in June 2018, which was replaced in May 2019. He experiences sporadic seizures, which are well-controlled (timeline of events in Table 1).

**Table 1:** Timeline of events

Date	Event
June 2018	VPS implantation
May 2019	Catheter replacement
July 2025	First anal catheter exit → manual reinsertion by the patient
October 2025	New anal exteriorization + catheter traction + foul-smelling fluid discharge
October 2025	10/15—Joint surgery: laparotomy + craniotomy, with catheter removal (distal portion) and sigmoidorrhaphy + removal of the proximal portion by craniotomy
October 2025	Fourth postoperative day, cephalic surgical wound (Kocher’s point on the right) with signs suggestive of infection; antibiotic therapy with ceftriaxone initiated
October 2025	10/30—15th postoperative day—hospital discharge recommended to continue antibiotic therapy for 14 days of use
November 2025	Outpatient follow-up 11/25—41st postoperative day: patient asymptomatic, wounds clean and dry, bowel movements preserved, no new complaints

### Clinical examination

Good general condition, normochromic, normohydrated, afebrile, eupneic in ambient air, and without evidence of respiratory or hemodynamic distress. Visible externalization of the distal portion of the VPS catheter through the anal canal (Fig. 1). Flat, flaccid abdomen with no signs of peritonitis at the time of initial assessment.

**Figure 1:**
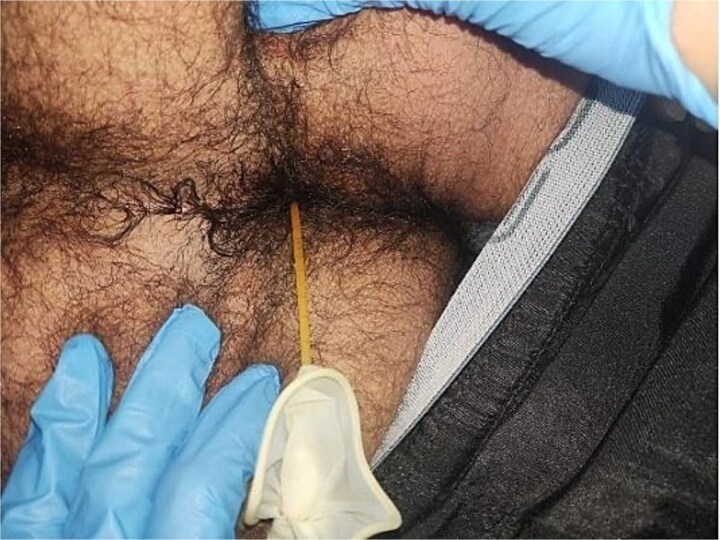
Externalized VPS catheter through the anal orifice.

### Imaging tests

Computed tomography (CT): visualization of the distal catheter in a topography compatible with the located in the intraparietal space of the abdominal wall (Fig. 2) and interior of the intestinal loop (Fig. 3). Findings suggestive of perforation of the digestive tract (Fig. 4) and intraluminal migration and anal exteriorization (Fig. 5).

**Figure 2:**
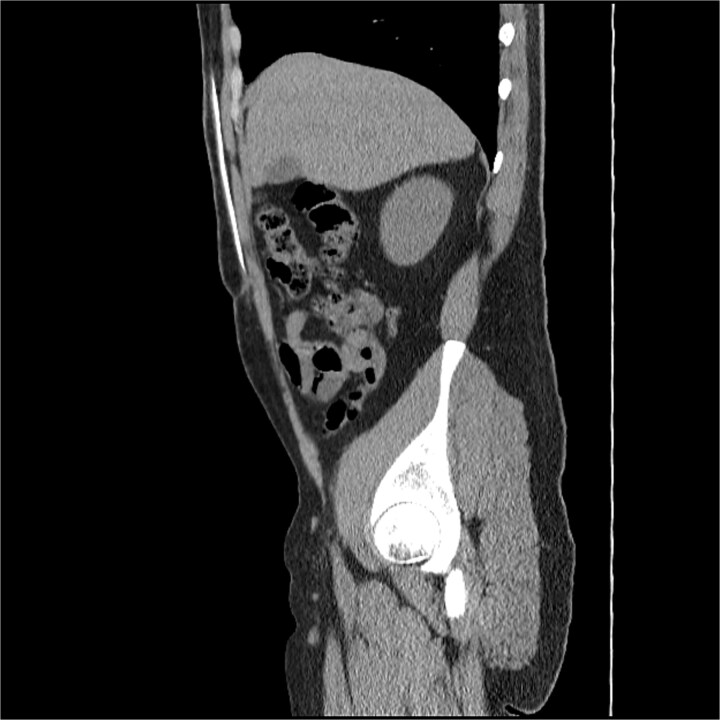
The catheter located in the intraparietal space of the abdominal wall before entering the abdominal cavity—sagittal section.

**Figure 3:**
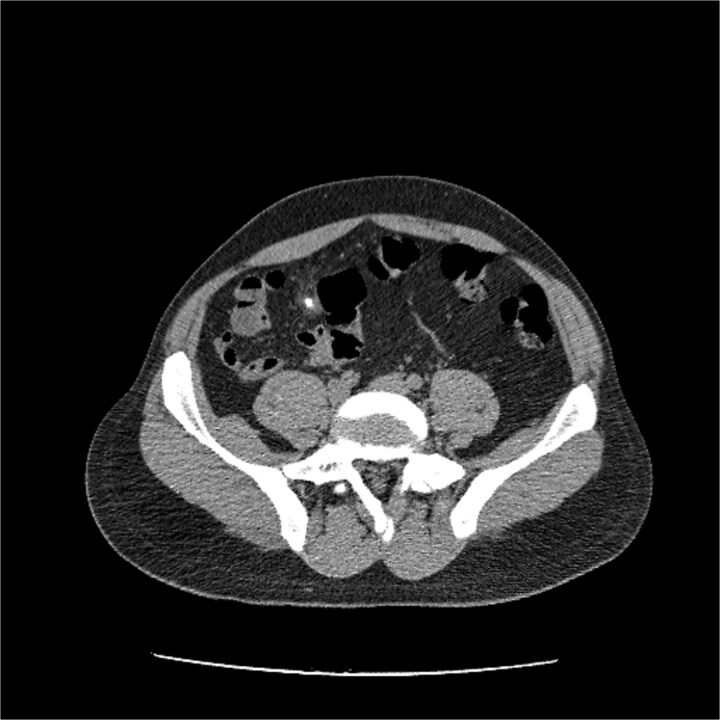
Hyperdense image showing the catheter inside the abdominal cavity before entering the sigmoid colon—axial section.

**Figure 4:**
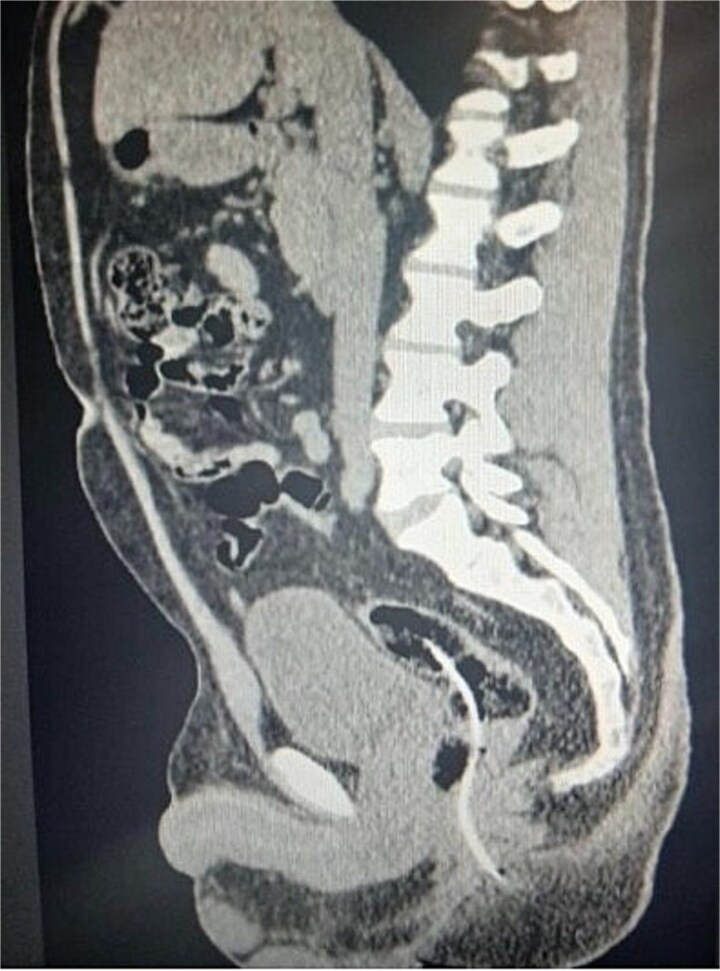
Tubular structure compatible with a catheter, following the path to the pelvic region and entering the intestinal loop (sigmoid colon), and an intraluminal path visible on CT—sagittal section.

**Figure 5:**
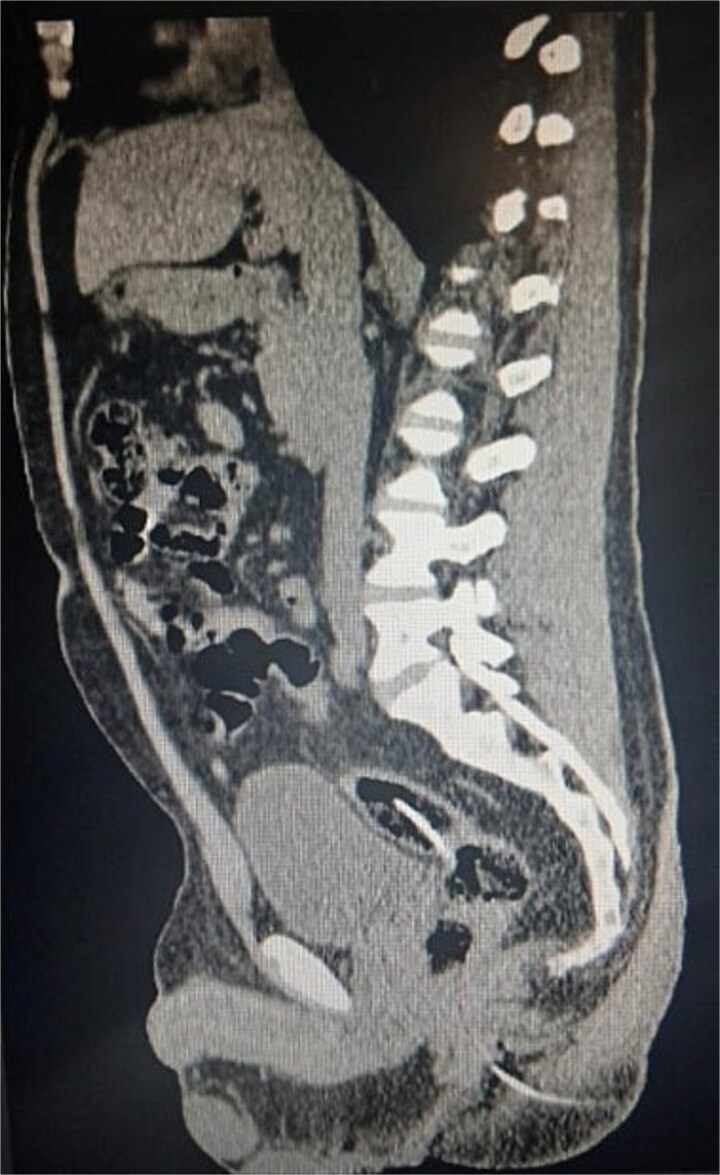
Tubular structure compatible with a catheter, protruding from the anal region as seen on CT—sagittal section.

### Diagnosis

Perforation of the gastrointestinal tract, in the sigmoid colon, with intraluminal migration and anal exteriorization of the distal end of the VPS catheter.

### Intervention

A multidisciplinary treatment approach was employed, involving collaboration among the neurosurgery, general surgery, and upper GI surgery teams. Surgical procedure: included exploratory laparotomy and craniotomy. During the procedure, firm adhesions of the sigmoid colon to the parietal peritoneum were identified, along with the intraluminal migration of the distal portion of the VPS catheter into the colon. The neurosurgery team was responsible for safely removing the proximal portion of the catheter via craniotomy, while the upper GI surgery team managed the removal of the distal portion. A median laparotomy incision was made, revealing no free fluid in the abdominal cavity. The distal catheter had migrated from the abdominal wall to the sigmoid colon, necessitating careful extraction and repair (Fig. 6).

**Figure 6:**
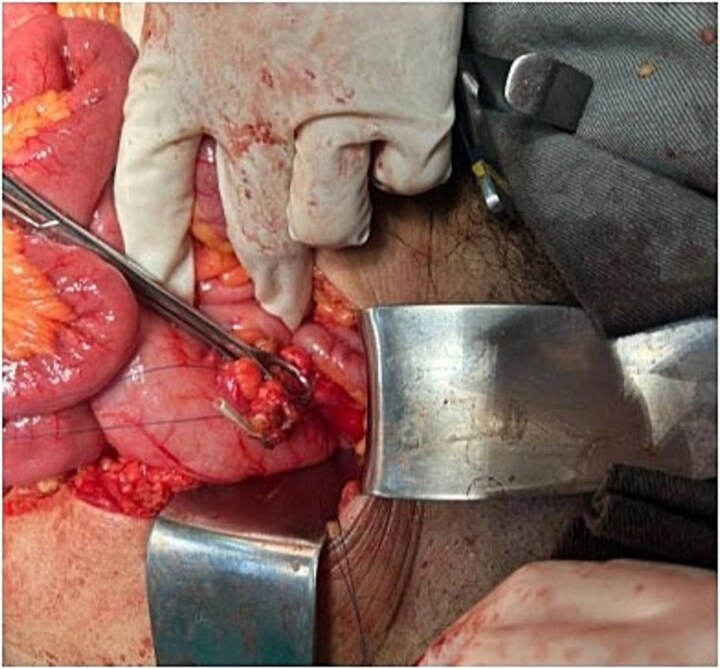
Surgical procedure: segment of the catheter already sectioned in the abdominal wall, visualized entering the sigmoid colon.

### Progression and outcome

The patient exhibited satisfactory immediate postoperative recovery, maintaining hemodynamic stability, adequate nutritional intake, and no signs of infection throughout the hospital stay. However, the cephalic surgical wound developed signs suggestive of infection on the fourth postoperative day, prompting empirical antibiotic therapy with ceftriaxone. Subsequent cultures of cerebrospinal fluid showed no signs of infection, and intravenous antibiotic therapy continued for a total of 14 days. The patient was discharged on 30 October 2025, on the 15th postoperative day, with recommendations for continued oral antibiotics for another 14 days and follow-up with the General Surgery and Neurosurgery teams. On the 41st postoperative day, he returned for outpatient follow-up, presenting in good general condition, asymptomatic, with clean and dry surgical wounds, normal bowel movements, and no complaints.

## Discussion

Intestinal perforation by a VPS catheter is a rare complication, and its pathophysiology often involves prolonged erosion of the intestinal wall by the catheter, as well as chronic local inflammatory reactions [4–6, 8]. Risk factors include multiple shunt revisions, prolonged catheter implantation time, and the clinical condition of the patient [2, 9]. The clinical presentation can range from asymptomatic anal exteriorization to severe cases requiring urgent intervention [5, 8, 10]. Timely diagnosis and management are crucial for preventing complications such as peritonitis and secondary neurological issues. A collaborative approach among surgical specialties is essential for optimal outcomes [6, 9, 10]. This case reinforces the significance of promptly recognizing intestinal perforation as a rare but serious complication of ventriculoperitoneal shunting. Multidisciplinary management and early recognition and removal of the catheter proved to be critical to the patient’s favorable outcome.
